# A Study of Canine Rocky Mountain Spotted Fever Prevalence Seen in a South Carolina Veterinary Lameness and Performance Referral Center

**DOI:** 10.3390/vetsci13060585

**Published:** 2026-06-16

**Authors:** Robert L. Gillette, Vijaya V. Indukuri, Jeannie Willems, Denise Passmore

**Affiliations:** 1 Department of Small Animal Clinical Sciences, College of Veterinary Medicine, Michigan State University, East Lansing, MI 48824, USA; 2 Nutramax Laboratories, 946 Quality Drive, Lancaster, SC 29720, USA; vindukuri@nutramaxlabs.com (V.V.I.); jdubs777.jw@gmail.com (J.W.); dpassmore@nutramaxlabs.com (D.P.)

**Keywords:** dog, canine, Rocky Mountain spotted fever, rickettsia, North Carolina, South Carolina, performance dog, working dog, disease prevalence

## Abstract

Rocky Mountain spotted fever (RMSF), caused by *Rickettsia rickettsii*, is a potentially severe tick-borne disease affecting both dogs and humans. While commercial in-clinic diagnostic tests have improved the detection of several common canine tick-borne diseases, RMSF is not routinely included in many point-of-care screening panels. In addition, infected dogs may present with subtle or nonspecific clinical signs, making diagnosis challenging. This retrospective observational study evaluated the occurrence of PCR-confirmed *Rickettsia rickettsii* infection in dogs presented to a veterinary lameness and performance referral center. From March 2022 through February 2024, a convenience sample of 51 dogs underwent RMSF testing as part of their diagnostic evaluation. Blood samples were analyzed using a commercial vector-borne disease PCR panel. Overall, 13 of 51 dogs (25.5%) were PCR-positive for Rickettsia rickettsii. Thirty-six dogs originated from North Carolina and South Carolina, of which 13 (36.1%) were PCR-positive. These findings suggest that RMSF should be considered among the differential diagnoses in dogs presenting with unexplained lameness, reduced athletic performance, or other nonspecific clinical concerns in regions where tick-borne diseases are common. The identification of PCR-confirmed Rickettsia rickettsii infection in this referral population highlights the potential value of expanded vector-borne disease testing.

## Simple Summary

Tick-borne diseases are a recognized health issue in the United States. The use of commercial, in-clinic test kits has greatly enhanced the diagnostic capabilities for several common tick-borne infections; however, Rocky Mountain spotted fever (RMSF) is not routinely included in many point-of-care screening panels. Many dogs infected with tick-borne pathogens may not exhibit typical clinical signs, and athletic or working dogs may present with subtle performance deficits rather than obvious illness. From 2022 through 2024, a convenience sample of 51 dogs evaluated at the Nutramax Wellness and Performance Center in Lancaster, South Carolina, underwent RMSF testing in addition to the IDEXX 4Dx Plus Test. Of these dogs, 16 originated from North Carolina, and 20 originated from South Carolina. Thirteen of the 36 dogs (36.1%) from the Carolinas were PCR-positive for Rickettsia rickettsii, the causative agent of RMSF. Overall, 13 of the 51 dogs (25.5%) tested positive. The findings from this study suggest that RMSF should be considered among the differential diagnoses in dogs presenting with unexplained lameness, reduced athletic performance, or other nonspecific clinical signs in regions where tick-borne diseases are common. Additional studies are warranted to better understand the clinical significance of PCR-confirmed RMSF infection in dogs.

## 1. Introduction

Tick-borne diseases (TBDs) are an important and growing health concern affecting both canine and human populations throughout the United States [1,2]. Changes in climate, wildlife populations, vector distribution, and increased awareness have contributed to the continued emergence and recognition of tick-borne pathogens in many geographic regions [1,2]. Commercial in-clinic diagnostic tests have substantially improved the ability of veterinarians to identify several common canine tick-borne diseases, including infections caused by *Ehrlichia* spp., *Anaplasma* spp., *Borrelia burgdorferi*, and *Dirofilaria immitis* [3,4,5]. Clinical manifestations associated with these infections can be highly variable and may include lethargy, fever, decreased appetite, weight loss, lymphadenopathy, lameness, joint pain, exercise intolerance, and other nonspecific signs [6,7]. However, some infected dogs may remain asymptomatic or exhibit only subtle clinical abnormalities, making diagnosis challenging.

Subclinical or minimally symptomatic tick-borne infections may be particularly relevant in athletic, working, and sporting dogs, where small changes in performance may be recognized before more obvious clinical signs develop. In racing greyhounds, approximately one-half of retired racing dogs evaluated as potential blood donors were reported to be positive for *Babesia canis* or *Ehrlichia canis* despite appearing clinically normal [8]. However, the primary author’s clinical experience suggested that subclinical tick-borne infections may have important implications for canine performance. In the early 1990s, some racing greyhounds presented with a history of repeatedly finishing out of the money and dropping two racing grades despite the absence of major musculoskeletal abnormalities. Following treatment with tetracycline, several of these dogs returned to racing with improved performance [9]. Although this response does not establish causation, it suggests that underlying tick-borne infections may have adversely affected the dogs’ health, metabolism, or performance despite the absence of overt clinical signs. These observations support the concept that dogs may harbor vector-borne pathogens without exhibiting obvious illness [10]. From a clinical perspective, subtle changes in performance, endurance, recovery, or musculoskeletal function may still be recognized by owners, trainers, and veterinarians, leading to increased interest in the potential impact of subclinical or minimally symptomatic tick-borne infections on athletic performance.

Rocky Mountain spotted fever (RMSF), caused by *Rickettsia rickettsii*, is a potentially severe tick-borne disease affecting both dogs and humans. *Rickettsia rickettsii* belongs to the spotted fever group rickettsioses and is transmitted primarily through infected ticks [11,12]. Following infection, the organism targets vascular endothelial cells, resulting in vasculitis and varying degrees of vascular injury. Consequently, clinical manifestations may range from mild or nonspecific illness to severe systemic disease characterized by fever, lethargy, neurologic abnormalities, edema, hemorrhage, multisystem organ involvement, and death [7,12,13]. The extent and severity of vascular injury contribute to the broad spectrum of clinical signs observed in affected animals. The primary author subsequently observed similar performance-related concerns in other working breeds, including pointing bird dogs, foxhounds, and retrievers, while practicing at the Animal Health and Performance Program, College of Veterinary Medicine, Auburn University. Dogs that tested positive for one of four common tick-borne diseases (*Ehrlichia* spp., *Anaplasma* spp., *Babesia* spp., or Rocky Mountain spotted fever) and presented with mild lameness, reduced performance, or other subtle clinical concerns often responded favorably to a course of doxycycline therapy. These responses were similar to those observed in racing greyhounds and suggest that tick-borne infections may have adversely affected the dogs’ health, metabolism, or athletic performance despite the absence of overt clinical signs. Although these observations do not establish a causal relationship, they support the possibility that subclinical or minimally symptomatic tick-borne infections may contribute to performance limitations in working and sporting dogs.

Diagnosis of RMSF can be challenging. While commercial point-of-care screening tests have improved recognition of several common canine vector-borne diseases, Rickettsia rickettsii is not routinely included in many commonly used in-clinic screening panels [3,4,5]. Definitive diagnosis often requires additional laboratory testing, including serology and molecular diagnostic techniques such as polymerase chain reaction (PCR) testing [14]. Furthermore, clinical signs associated with RMSF frequently overlap with those associated with other vector-borne pathogens, including *Ehrlichia* spp., *Anaplasma* spp., *Babesia* spp., *Borrelia* spp., and *Bartonella* spp. [7,15,16,17]. As a result, RMSF may be underrecognized in dogs presenting with nonspecific clinical signs, lameness, or reduced athletic performance.

North Carolina and surrounding southeastern states have historically reported relatively high numbers of human RMSF cases, suggesting continued regional exposure risk to Rickettsia-infected tick populations [11]. Although RMSF has been well described in both veterinary and human medicine, limited information is available regarding its occurrence in dogs referred for evaluation of lameness, exercise intolerance, or unexplained performance deficits. During clinical practice at the Animal Health and Performance Program at Auburn University and subsequently at the Nutramax Wellness and Performance Center (NWPC), the primary author observed that some dogs presenting with unexplained lameness or performance concerns were subsequently identified as having tick-borne infections. These observations prompted further investigation into the potential occurrence of RMSF within this referral population.

The objective of the present study was not to determine the prevalence of RMSF within the general canine population, but rather to describe the occurrence of PCR-confirmed Rickettsia rickettsii infection in dogs referred to a veterinary lameness and performance center. Specifically, this retrospective observational study evaluated dogs presented to the NWPC for lameness, musculoskeletal complaints, or performance-related concerns that underwent additional RMSF testing as part of a comprehensive vector-borne disease diagnostic evaluation.

## 2. Materials and Methods

The data for this retrospective observational study were compiled using test results from dogs presented to the NWPC, Lancaster, SC. The focus of the NWPC was to gather information on dogs with hard-to-diagnose lameness or athletic and working dogs described as having performance issues. The dogs were referred to the center by their veterinarians. When the dogs and clients arrived at the NWPC, the clients were informed that the focus of the NWPC was to collect related data, and they were given a brochure describing the goals. The clients signed a patient data form acknowledging that the information collected at the center would be used for study and publication purposes. The director of the NWPC was a veterinarian and diplomate of the American College of Veterinary Sports Medicine and Rehabilitation. If testing revealed a musculoskeletal issue that could be resolved, treated, or managed, the therapeutic plan was presented to the owner. If the patient had a non-surgical issue that could be resolved or partially resolved onsite at the time of the visit, treatment was performed, or a management plan was initiated. The patient and client were then sent home to be managed further by their veterinarian.

From 1 March 2022 through 29 February 2024, a convenience sample of 51 dogs was tested for RMSF at NWPC, Lancaster, South Carolina, in addition to receiving the IDEXX 4Dx Plus Test. For RMSF testing, blood was drawn from the jugular vein using a 12-cc syringe with a 20–21 g × 1” needle. 

Blood samples were collected into EDTA and serum collection tubes and shipped to a commercial diagnostics laboratory for analysis. The IDEXX 4Dx Plus Test was performed with one set of samples, while a second set was submitted to Antech Diagnostics for vector-borne disease testing. Rocky Mountain spotted fever testing was included within a canine vector-borne disease panel. The panel detects 16 infectious agents associated with canine vector-borne diseases through a combination of real-time polymerase chain reaction (PCR) testing for 15 pathogens and serologic detection of antibodies to Borrelia burgdorferi. All positive RMSF cases reported in this study were PCR-positive for *Rickettsia rickettsii*.

## 3. Results

A total of 51 dogs were evaluated between March 2022 and February 2024 and underwent RMSF testing as part of a comprehensive vector-borne disease diagnostic evaluation. The dogs originated from 11 states across the United States; however, the majority of dogs (36/51; 70.6%) originated from North Carolina and South Carolina (Table 1).

Overall, 13 of the 51 dogs (25.5%) were PCR-positive for Rickettsia rickettsii. All positive cases originated from either North Carolina or South Carolina. Among the dogs originating from the Carolinas, 13 of 36 dogs (36.1%) tested PCR-positive for Rickettsia rickettsii. No dogs originating from outside North Carolina or South Carolina tested positive for RMSF during the study period (Table 1).

The study population consisted of a variety of breeds, reflecting the diverse referral population evaluated at the Nutramax Wellness and Performance Center. Whippets represented the most common breed enrolled in the study, accounting for 17 of 51 dogs (33.3%) (Table 2). Positive dogs were distributed across multiple breed types, with PCR-positive cases identified in 12 different breeds. Labrador retrievers were the only breed represented by more than one positive dog. No obvious breed-specific pattern was observed within the study population; however, the sample size was not sufficient to formally evaluate breed-associated risk factors.

The distribution of dogs by state of origin and PCR-positive RMSF status is presented in Table 1, while the breed distribution of enrolled dogs and PCR-positive cases is summarized in Table 2.

## 4. Discussion

This retrospective observational study identified PCR-confirmed Rickettsia rickettsii infection in 13 of 51 dogs evaluated at a veterinary lameness and performance referral center. Among dogs originating from North Carolina and South Carolina, 13 of 36 dogs (36.1%) were PCR-positive. Although the study population consisted of a highly selected referral population and should not be considered representative of the general canine population, these findings suggest that RMSF should be considered among the differential diagnoses in dogs presenting with unexplained lameness, reduced performance, or other nonspecific clinical concerns in regions where tick-borne diseases are common [1,2,7].

The positive dogs represented multiple breeds, with no apparent breed predilection observed within this study population. Positive cases were identified in 12 different breed types, with Labrador Retrievers being the only breed represented by more than one positive dog. However, the relatively small sample size and referral-based nature of the study preclude a meaningful assessment of breed-associated risk factors. Future investigations involving larger populations are required to determine whether breed-specific differences in susceptibility exist.

An important consideration when interpreting RMSF diagnostic testing is the distinction between exposure, infection, and clinical disease. Positive serologic testing may reflect previous exposure, prior infection, or current infection, making interpretation difficult in endemic regions where repeated tick exposure is common [12,14]. In contrast, all positive cases identified in the present study were confirmed through PCR-based detection of *Rickettsia rickettsii* DNA. PCR positivity provides stronger evidence of active infection than serology alone because it demonstrates the presence of pathogen genetic material within the patient sample [14,18]. Nevertheless, detection of pathogen DNA does not independently establish that RMSF was the direct cause of the presenting complaint. Clinical findings, patient history, physical examination results, and additional diagnostic information should always be considered when interpreting PCR-positive results [7,14].

The clinical significance of PCR-confirmed Rocky Mountain spotted fever (RMSF) in dogs presenting with lameness or reduced athletic performance remains incompletely understood. The primary author has observed that some athletic and working dogs with subtle performance deficits and positive tick-borne disease test results appeared to improve following treatment with tetracycline-class antibiotics. Similar observations have been reported anecdotally by veterinarians working with performance and sporting dogs. However, because the present study was retrospective and did not include standardized treatment protocols, objective outcome measurements, or untreated control groups, no conclusions regarding causality or treatment efficacy can be drawn. Consequently, the findings reported here should be interpreted as an association between PCR-confirmed RMSF and the evaluated referral population rather than evidence that RMSF was the primary cause of the observed clinical signs or performance deficits. Prospective, controlled studies are required to determine whether subclinical or minimally symptomatic RMSF infection contributes to reduced performance in athletic and working dogs and whether antimicrobial treatment results in measurable clinical improvement [7,13].

Another important consideration is the potential contribution of other vector-borne pathogens. Dogs residing in endemic regions may be exposed to multiple infectious agents, including *Ehrlichia* spp., *Anaplasma* spp., *Babesia* spp., *Borrelia* spp., and *Bartonella* spp. [7,15]. Several of these pathogens can produce overlapping clinical manifestations, including lethargy, musculoskeletal discomfort, exercise intolerance, lameness, fever, and nonspecific performance deficits [7,15,16]. *Bartonella* spp. are of particular interest because infection has been associated with chronic inflammatory conditions, lameness, endocarditis, and a variety of poorly defined clinical syndromes in dogs [15,16,17]. Furthermore, Bartonella testing is not routinely included in many in-clinic screening platforms [15]. Therefore, the presence of PCR-confirmed RMSF does not exclude the possibility of coinfection or concurrent disease processes contributing to the clinical presentation. Future studies incorporating broader vector-borne disease panels may provide additional insight into the relative contribution of multiple infectious agents in dogs with unexplained performance limitations or lameness.

Several limitations should be considered when interpreting the findings of this study. First, the study population consisted of a convenience sample of dogs referred to a veterinary lameness and performance center and, therefore, may not be representative of the general canine population in North Carolina, South Carolina, or other regions of the United States. Referral populations frequently differ from primary care populations with respect to disease prevalence, clinical complexity, and owner awareness. Consequently, the frequency of PCR-positive RMSF cases observed in this study should not be interpreted as a population prevalence estimate [1,6]. Second, because this investigation was retrospective in nature, complete demographic and clinical information was not available for all dogs. Variables such as travel history, tick prevention status, duration of clinical signs, concurrent medical conditions, and exposure risk factors could not be consistently evaluated. Third, the relatively small sample size limited statistical analysis and prevented meaningful evaluation of breed, age, sex, or geographic risk factors.

Despite these limitations, the findings suggest that RMSF should be considered among the differential diagnoses in dogs presenting with unexplained lameness, exercise intolerance, or reduced athletic performance, particularly in regions where tick exposure is common [7,11,13]. The identification of 13 PCR-confirmed cases among 51 dogs evaluated at a specialty referral center highlights the potential value of expanded vector-borne disease testing when routine diagnostic evaluation fails to identify an underlying cause. Prospective studies involving larger populations, standardized clinical assessments, longitudinal follow-up, and comprehensive vector-borne disease screening are warranted to better define the occurrence, clinical significance, treatment response, and long-term outcomes associated with PCR-confirmed RMSF infection in dogs.

## 5. Conclusions

Rocky Mountain spotted fever (RMSF) remains an important tick-borne disease of veterinary and public health significance [2,7,13]. In this retrospective observational study, 13 of 51 dogs (25.5%) evaluated at a veterinary lameness and performance referral center were PCR-positive for Rickettsia rickettsii, including 13 of 36 dogs (36.1%) originating from North Carolina and South Carolina. These findings suggest that RMSF should be considered among the differential diagnoses in dogs presenting with unexplained lameness, reduced athletic performance, or other nonspecific clinical concerns in regions where tick-borne diseases are endemic [7,11,13].

Because all positive cases were identified through PCR-based detection, the findings provide evidence of infection rather than serologic evidence of prior exposure alone [14,18]. However, PCR positivity does not independently establish causality for the presenting complaint, and results should be interpreted in conjunction with clinical findings, patient history, and other diagnostic information [7,14].

Although no vaccine is currently available for RMSF, early recognition and appropriate diagnostic evaluation remain important components of clinical management [13,14,19,20]. The findings from this study support consideration of expanded vector-borne disease testing in dogs with unexplained clinical signs when routine diagnostic investigations fail to identify an underlying cause [1,2,7]. Additional prospective studies are warranted to better define the clinical significance, treatment response, and long-term outcomes associated with PCR-confirmed Rickettsia rickettsii infections in dogs [1,2,14,18].

## Figures and Tables

**Table 1:** Dogs sampled per state and diagnosed with RMSF.

State	2022	2023	2024	Total	RMSF
Florida	2			2	
Georgia	2	1		3	
GA/FL Border			1	1	
Illinois	1	1		2	
Indiana	1			1	
North Carolina	4	8	4	16	6
New Jersey		1		1	
New York	1	2		3	
South Carolina	9	8	3	20	7
Tennessee		1		1	
Washington	1			1	
**Total**	21	22	8	51	13

**Table 2:** Dog breeds sampled and diagnosed with RMSF.

Breed	Total	RMSF
Australian Shepherd	2	
Basenji	1	
Belgian Malinois	1	
Border Collie	3	1
Borzoi	1	1
Boxer	1	1
Carolina Dog	1	
Chesapeake Bay Retriever/Labradoodle	1	1
Doberman Pinscher	1	1
English Bulldog	1	
German Shorthair Pointer	4	1
German Wirehaired Pointer	1	1
Golden Retriever	1	
Great Pyrenees	1	1
Greyhound	3	
German Shepherd	1	
Labrador Retriever	3	2
Mixed Breed	1	
Pitbull	1	1
Pitbull Mix	1	
Rhodesian Ridgeback	1	
Vizsla	2	1
Whippet	17	1
Whippet Mix	1	
**Total**	51	13

## Data Availability

The original contributions presented in this study are included in the article. Further inquiries can be directed to the corresponding author.

## Abbreviations

The following abbreviations are used in this manuscript:

TBD	Tick-borne disease
RMSF	Rocky Mountain spotted fever
NWPC	Nutramax Performance and Wellness Center
EDTA	Ethylenediaminetetraacetic acid
PCR	Polymerase chain reaction
